# Development of an exploratory prediction model for preoperative CK19 expression in esophageal cancer driven by radiomics and machine learning

**DOI:** 10.1371/journal.pone.0350744

**Published:** 2026-06-01

**Authors:** Jiafu Yang, Sicheng Wang, Xin Chen, Liping Liu, Yangkang Li

**Affiliations:** 1 Department of Radiology, Guangdong Engineering Research Center of AI-Powered Precision Cancer Diagnostics and Therapeutics(Proposed)/GuangDong Engineering Technology Research Center of AI-Powered Precision Cancer Diagnostics and Therapeutics, Cancer Hospital of Shantou University Medical College, Shantou, China; 2 University of Macau, Macau, China; University of Pisa, ITALY

## Abstract

**Background:**

Esophageal cancer ranks among the most lethal malignancies worldwide, particularly prevalent in the Guangdong–Chaoshan region of China due to regional dietary habits. Cytokeratin 19 (CK19) is an important immunohistochemical marker reflecting tumor invasiveness and metastatic potential; however, noninvasive preoperative prediction of CK19 expression remains unavailable. This study aimed to develop a CT-based radiomics model combined with machine learning to predict CK19 expression preoperatively.

**Methods:**

This study included 134 patients with primary esophageal cancer. All patients underwent enhanced CT scans before surgery, and CK19 expression was evaluated by pathological analysis after surgery. Radiomics technology was used to extract multidimensional image features including shape, texture, and first-order features from CT images. A prediction model was established by combining machine learning models such as gradient boosted decision tree (GBDT), random forest (RF), extreme gradient boosting (XGB), and lightweight gradient boosting machine (LGBM), and the interpretability of the model was analyzed by SHAP value.

**Results:**

The random forest model showed relatively higher accuracy and precision among the compared models, with an AUC value of 0.6765 and an accuracy of 0.8293. GBDT demonstrated a more balanced performance (AUC: 0.6597), while XGB (AUC: 0.6744) and LGBM (AUC: 0.6807) showed comparable but overall slightly lower discriminative ability. Feature importance analysis showed that the features after wavelet transformation made a significant contribution to the prediction results. The results verified the potential of radiomics combined with machine learning technology in the preoperative prediction of CK19 expression.

**Conclusion:**

This study developed a preoperative noninvasive prediction model based on radiomics and machine learning, which showed modest predictive performance in an exploratory setting in the evaluation of CK19 markers in patients with esophageal cancer, may provide preliminary support for further exploration in precision medicine. In the future, the clinical applicability of this model needs to be further verified and its promotion and application in a larger population needs to be optimized.

## 1 Introduction

Esophageal carcinoma, ranking as the eighth most prevalent cancer worldwide and the sixth leading cause of cancer-related mortality, exhibits stark epidemiological disparities [1]. In particular, the Guangdong Chaoshan area of China records disproportionately high incidence rates, influenced significantly by local dietary customs, including a preference for drinking hot tea [1]. Despite advances in medical treatments, the overall prognosis for esophageal cancer remains grim, with most cases identified at advanced stages [2]. The evaluation of disease status and future risk mainly utilizes advanced imaging modalities—computed tomography (CT), magnetic resonance imaging (MRI), and positron emission tomography (PET)—alongside immunohistochemical techniques to detect essential molecular biomarkers [2]. Nonetheless, these diagnostic approaches are limited by their potential for human error, interpretative difficulties, and lack of consistency, critically restricting their utility in clinical practice. Thus, there is an urgent need to refine the precision and reliability of these techniques to improve prognostic determinations and enable the development of personalized therapeutic strategies for esophageal cancer.

To address these limitations, recent studies have increasingly focused on quantitative imaging biomarkers to improve the evaluation of upper gastrointestinal tumors. These imaging biomarkers are quantitative indicators extracted from CT or MRI that reflect tumor microstructural heterogeneity and biological behavior, providing objective and reproducible information beyond routine visual interpretation [3]. Imaging biomarker research has demonstrated that such quantitative features correlate with clinically relevant outcomes in upper GI cancers, offering important methodological support for the development of radiomics in esophageal cancer. Within esophageal cancer, multiple radiomics investigations have reported encouraging results: CT-based radiomic signatures have shown value in predicting lymph node metastasis, radiomics-clinical models have enhanced the assessment of therapeutic response following neoadjuvant treatment, and texture-based approaches have contributed to more refined survival stratification [4,5]. These findings indicate that quantitative features extracted from standard imaging can capture biological information not fully appreciable on conventional assessment. However, despite these advances, existing radiomics studies have largely centered on staging or treatment response, with limited exploration of whether imaging-derived features can non-invasively reflect molecular phenotypes or immunohistochemical markers in esophageal cancer.

In the context of esophageal cancer, immunohistochemical markers such as HER2, PD-L1, p53, and cytokeratin 19 (CK19) provide clinically relevant information for tumor characterization, therapeutic stratification, and risk assessment. HER2 has been associated with aggressive tumor behavior and targeted therapeutic strategies, while PD-L1 is closely related to immunotherapy response, and p53 alterations have been linked to poorer prognosis and treatment resistance [6–9]. In addition to these widely studied markers, cytokeratin 19 (CK19) is an epithelial intermediate filament protein that can help characterize the epithelial phenotype of esophageal tumors and provide complementary immunohistochemical information for pathological assessment [10,11]. Beyond its diagnostic relevance, CK19 has also been investigated in relation to tumor progression and metastatic behavior in esophageal squamous cell carcinoma. Previous studies have suggested that cytokeratin expression and distribution patterns involving CK19 may be associated with disease progression, lymph node metastasis, and prognosis in esophageal squamous cell carcinoma [12,13].

Given the potential relevance of CK19 to epithelial phenotype characterization, tumor progression, and risk assessment in esophageal cancer, leveraging advanced imaging techniques and machine learning may provide a feasible approach for non-invasively estimating CK19 expression. This study aims to develop an exploratory radiomics-based model for predicting CK19 expression using machine learning approaches applied to preoperative CT images.By extracting and analyzing quantitative imaging features, this research seeks to bridge the gap between imaging data and molecular marker prediction, improving diagnostic precision and enabling more tailored therapeutic strategies for esophageal cancer patients. Through this innovation, we aspire to provide a robust, interpretable tool that integrates seamlessly into clinical workflows, ultimately contributing to the advancement of personalized medicine in oncology.

## 2 Method

### 2.1 Study cohort and dataset

**Fig 1:**
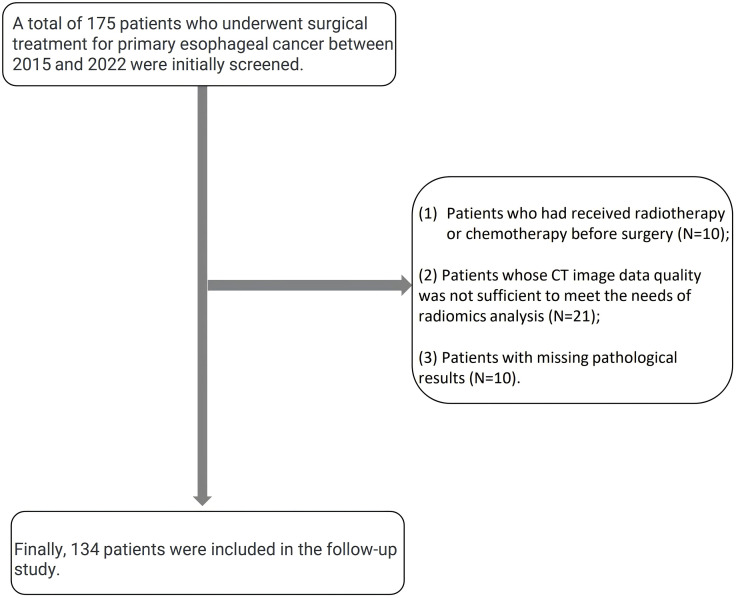
Flow chart of patient enrollment in this study.

This retrospective study came from Shantou University Medical College Cancer Hospital. We conducted this study after obtaining approval from the institutional medical ethics committee (ethics number: 2023116). Since this study was retrospectively designed and data collection was based on existing medical records, written informed consent was waived. The study initially screened 175 patients who underwent surgical treatment for primary esophageal cancer between 2015 and 2022. All patients completed enhanced CT scans before surgery, and the expression of the immunohistochemical marker cytokeratin 19 (CK19) in tumor tissue was evaluated by pathological examination after surgery. The inclusion criteria for patients were: (1) a clear diagnosis of primary esophageal cancer by pathology; (2) complete preoperative CT imaging data; (3) complete postoperative pathological and immunohistochemical analysis reports. Exclusion criteria included: (1) patients who had received radiotherapy or chemotherapy before surgery; (2) CT imaging data quality was not sufficient to meet the needs of radiomics analysis; (3) patients with missing pathological results. The samples in this study represent the patient population with typical esophageal cancer characteristics in this institution, providing a reliable data basis for model development. The patient enrollment process is shown in Fig 1, and 134 patients were finally included in the follow-up study.

### 2.2 Imaging protocol

Preoperative CT scans in this study were performed using two CT scanners: a GE BrightSpeed 16-row scanner (GE HealthCare, Boston, USA) and a Philips Ingenuity 64-row scanner (Philips HealthCare, Amsterdam, Netherlands). The scan range was from the hypopharynx to the lower edge of the diaphragm to ensure full coverage of the esophagus and its adjacent structures. The scans were performed using spiral CT technology, with the slice thickness and interslice spacing set to 5 mm, the tube current automatically adjusted within 160–300 mA, and the tube voltage fixed at 120 kVp. The rotation time was 0.75 s per revolution, the pitch was set to 0.999, and the single scan time was approximately 7.5 s.

Before scanning, patients fasted for at least 6 hours and drank 600 mL of water in divided doses within 15–20 minutes before the examination to increase gastrointestinal contrast; an additional 200 mL of water was drunk immediately before the scan. During scanning, patients were positioned supine with their hands raised and crossed above the head and were fixed with an elastic band to reduce motion artifacts. All patients were instructed to take a deep breath and hold it to complete the scan in a single breath-hold.

Enhanced scans were performed using a bolus-tracking technique. A nonionic contrast agent (iopromide, 300 mg I/mL; Ultravist, Bayer, Germany) was injected intravenously at a rate of 2.5 mL/s, with a dose of 1.5 mL per kilogram of body weight (typically 75–100 mL), followed by a 30 mL saline flush. The region of interest (ROI) was placed in the thoracic aorta, and scanning was automatically triggered when the attenuation reached 150 HU, with a 3-second delay after the trigger to ensure optimal vascular enhancement and lesion contrast.

### 2.3 Tumor segmentation

This study selected venous phase enhanced CT images as the basis for manual segmentation of the region of interest (ROI) because it performs best in contrast enhancement between tumors and surrounding tissues and can significantly improve the recognition of tumor boundaries. All segmentation work is based on the original image files in DICOM format and is completed through the dedicated medical image processing software 3D Slicer. During the segmentation process, the largest lesion region (Largest Tumor Region, LTR) of the tumor is marked first. For patients with a single tumor, the complete lesion outline is directly outlined; for patients with multiple lesions, the largest lesion region that is representative and available for subsequent analysis is selected based on the size and location of the lesion. The main purpose of this strategy is to ensure that the segmented region represents the overall characteristics of the lesion to the greatest extent possible, while reducing the impact of multi-lesion segmentation on the complexity of the subsequent model.

To ensure the accuracy and consistency of the segmentation results, this study adopted a two-person blind segmentation process. The initial segmentation was completed by a radiologist with 5 years of imaging diagnosis experience, and the segmentation results were mainly based on the clarity of the tumor boundary on the image and the contrast characteristics with the surrounding tissue. Subsequently, another senior radiologist with 20 years of rich experience independently reviewed the initial segmentation results and made necessary adjustments to the segmentation boundaries based on anatomical characteristics, imaging characteristics, and his own clinical experience. Finally, all segmentation results were finalized through consensus discussion between the two doctors to eliminate possible subjective biases in the segmentation process. To adapt to subsequent imaging omics analysis, all segmented ROIs were saved as standardized mask files and registered with the original CT images to ensure data consistency and traceability.

### 2.4 Feature extraction and screening

In order to extract reliable radiomics features from esophageal cancer CT images, this study used Pyradiomics, a feature extraction tool widely used in medical image analysis. For each preprocessed CT image, we extracted features covering multiple aspects such as shape, texture, and signal intensity, including first-order statistics, shape features, gray-level co-occurrence matrix (GLCM), gray-level size region matrix (GLRLM), gray-level dependency matrix (GLDM), and gray-level run length matrix (GLSZM). These multi-dimensional imaging features provide a rich data foundation for subsequent analysis.

The feature screening process includes several key steps. The intraclass correlation coefficient (ICC) is used to evaluate feature stability. Features with an ICC value of more than 0.75 are considered stable and can be included in subsequent analysis. Features with high ICC values are Z-score standardized to eliminate dimensional and order of magnitude differences between features. Features significantly associated with disease status were screened by t-test, and features with p values less than 0.05 were statistically significant. The Lasso regression model was used to streamline the feature range, and L1 regularization was used to complete feature selection, simplify the model, and improve prediction accuracy. The regularization parameters were optimized through cross-validation to screen the features that contributed most to the prediction model. These features are used for machine learning model training to build an efficient and accurate preoperative non-invasive prediction model for CK19 expression.

### 2.5 Machine learning prediction model

In order to accurately predict CK19 markers non-invasively before surgery, this study used four mainstream machine learning models, including gradient boosting decision tree (GBDT), random forest (RF), extreme gradient boosting (XGB), and lightweight gradient boosting machine (LGBM). Each model was optimized and validated under a series of predefined parameters.

#### 2.5.1 Random forest.

Random Forest (RF) is a powerful ensemble learning algorithm, especially suitable for processing high-dimensional data. The parameters of the RF model are set as follows: ` n_estimators = 100`, which means generating 100 decision trees; ` max_features = ‘sqrt’`, which means the number of randomly selected features at each split is the square root of the total number of features; ` min_samples_split = 2`, which means the minimum number of samples required for node splitting is 2; ` min_samples_leaf = 1`, which means the minimum number of samples contained in each leaf node is 1.

#### 2.5.2 Gradient boosting.

Gradient Boosting (GBDT) improves the overall performance of the model by gradually building a series of weak learners. The parameters of the GBDT model are set as follows: ` n_estimators = 100`, which means generating 100 weak learners; ` learning_rate = 0.1`, which means the weight update step size of each learner; ` max_depth = 3`, which means the maximum depth of each tree is 3; `subsample = 1.0`, which means using all samples for training.

#### 2.5.3 Extreme gradient boosting.

Extreme Gradient Boosting (XGB) is an optimized distributed gradient boosting library designed for high efficiency, flexibility, and portability. The parameters of the XGB model are set as follows: n_estimators = 100, which means generating 100 trees; learning_rate = 0.1, which controls the contribution of each tree; max_depth = 3, which limits the maximum depth of each tree; subsample = 1.0, which specifies the fraction of samples used for training each tree.

#### 2.5.4 Lightweight gradient boosting machine.

Lightweight Gradient Boosting Machine (LightGBM, LGBM) is an efficient gradient boosting framework designed specifically for large-scale datasets with faster training speed and lower memory consumption. LGBM improves computational efficiency through a histogram-based decision tree learning algorithm and a leaf-growing tree structure. The main parameter settings of the LGBM model are as follows: ‘n_estimators = 100’, which means generating 100 trees; ‘learning_rate = 0.1’, which controls the contribution of each tree; ‘max_depth = −1’, which means that the maximum depth of the tree is not limited; ‘num_leaves = 31’, which means that each tree has a maximum of 31 leaf nodes.

### 2.6 Statistical analysis

In this study, a variety of machine learning models were comprehensively evaluated and compared, aiming to develop an efficient model that can non-invasively predict CK19 marker expression before surgery. The experimental data were randomly divided into training and test sets in a ratio of 7:3. The training set was used for model construction and parameter optimization, while the test set was used to independently verify the predictive performance of the model to ensure the generalization ability of the model on unknown data. For the performance evaluation of the model, multiple indicators such as accuracy, recall, precision, F1 score, and area under the receiver operating characteristic curve (AUC) were used. In addition, the specific performance of the model under different classification categories was analyzed in detail through the confusion matrix to reveal potential misclassification patterns. In order to further explain the prediction mechanism of the model and improve its clinical interpretability, the SHAP (SHapley Additive exPlanations) value analysis tool was introduced. Based on the principles of game theory, SHAP can quantify the contribution of each feature to the model output. In this exploratory analysis, performance metrics are reported as point estimates without bootstrap confidence intervals or formal statistical comparisons, consistent with the hypothesis‑generating nature of the study.

## 3 Results

### 3.1 Experimental setup

The experiments in this study were completed on a high-performance computing workstation running the Windows 10 operating system, equipped with an Intel Core i9 processor and an NVIDIA GeForce RTX 3080 graphics card to ensure computational efficiency for model training and validation. All data processing and analysis used the Python 3.7 programming language, combined with a variety of open source libraries, including Scikit-learn, XGBoost, LightGBM, Pandas, and Numpy, to automate the entire process of data preprocessing, feature extraction, model training, and performance evaluation. In the experiment, radiomics features were extracted from standardized CT image masks, and the data were divided into training and test sets (7:3 ratio) under stratified random sampling to ensure that the proportion of CK19-positive and -negative cases in different groups was consistent to reduce the impact of sample distribution bias on the model.

### 3.2 Feature extraction and screening results

In this study, a total of 993 radiomics features were extracted from preoperative enhanced CT images of 134 patients, including 320 features from the original images and 673 features generated by wavelet transform. The original image features encompass multiple families, including shape, first‑order statistics, gray‑level co‑occurrence matrix (GLCM), gray‑level run length matrix (GLRLM), gray‑level size zone matrix (GLSZM), gray‑level dependence matrix (GLDM), and neighborhood gray‑tone difference matrix (NGTDM). The wavelet‑transformed features similarly cover these families across different frequency subbands, capturing tumor information at multiple scales and frequency domains.

**Fig 2:**
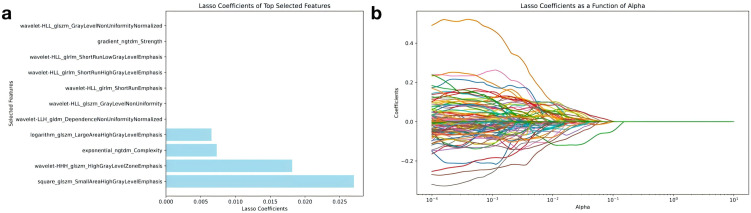
(a) Shows the key radiomics features selected by Lasso regression and their contributions, and (b) describes the dynamic changes of feature coefficients under changes in regularization strength (α value).

In order to reduce feature redundancy and screen the features that contribute most to model prediction, this study uses the Lasso regression method. By introducing the L1 regularization constraint, Lasso regression can compress the weights of unimportant features to zero, achieve feature sparsification and optimize model performance. After preliminary screening, a total of 72 features showed significant contributions, and then the regularization parameters were further optimized through cross-validation, and finally 11 most critical features were screened out. Fig 2a shows the Lasso coefficient ranking of these key features, among which wavelet- HLL_glszm_GrayLevelNonUniformityNormalized, logarithm_glszm_LargeAreaHighGrayLevelEmphasis, exponential_ngtdm_Complexity and other features have a significant influence in the prediction model. Fig 2b shows the change of Lasso coefficient with regularization strength (α value). As the α value increases, most feature coefficients gradually tend to zero, and only a small number of features that contribute significantly to the model are retained.

### 3.3 Preoperative non-invasive prediction of CK19 expression results

This study compared the performance of four mainstream machine learning models (GBDT, RF, XGB, and LGBM) in preoperative noninvasive prediction of CK19 expression, and the results are shown in Table 1. Among the four models, random forest (RF) showed relatively higher accuracy and precision, with an accuracy of 0.8293, a precision of 0.6824, a recall of 0.6134, an F1 score of 0.6325, and an AUC value of 0.6765. The gradient boosted decision tree (GBDT) was slightly inferior to RF in accuracy (0.7805) and recall (0.6408), but its F1 score (0.6328) was slightly higher, showing a better balanced performance. The performance of extreme gradient boosting (XGB) and lightweight gradient boosting machine (LGBM) was relatively close, both showing lower precision and recall (both 0.5167 and 0.5126), and AUC values of 0.6744 and 0.6807, respectively.

**Table 1:** Prediction results of various machine learning models in the validation set.

model	Accuracy	Precision	Recall	F 1-score	AUC
GBDT	0.7805	0.6269	0.6408	0.6328	0.6597
RF	0.8293	0.6824	0.6134	0.6325	0.6765
XGB	0.7561	0.5167	0.5126	0.5119	0.6744
LGBM	0.7561	0.5167	0.5126	0.5119	0.6807

Given the class imbalance in the dataset (83% CK19-negative vs. 17% CK19-positive), we additionally report balanced accuracy and Matthews correlation coefficient (MCC) to provide a more robust evaluation. For the random forest model, balanced accuracy was 0.613 and MCC was 0.288, indicating modest discriminative ability that is consistent with the AUC value.

**Fig 3:**
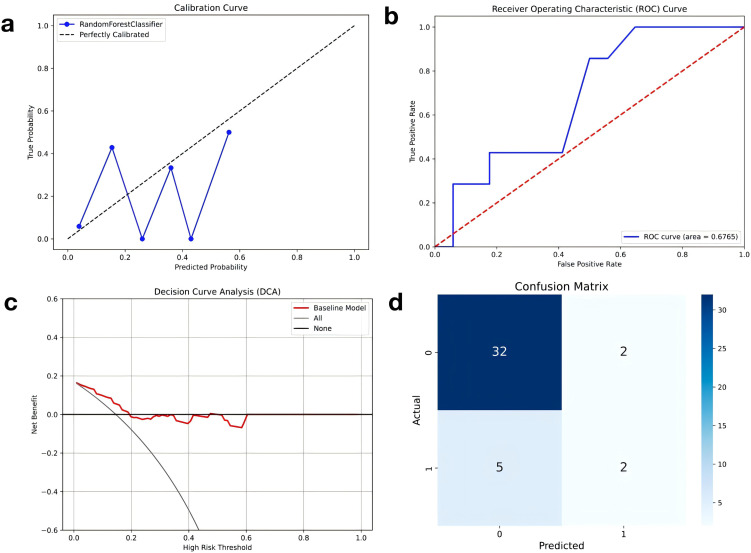
(a) The model calibration curve shows the comparison between the predicted probability and the actual probability, which is used to evaluate the calibration performance of the model. **(b)** The ROC curve describes the classification ability of the model at different decision thresholds, and the AUC value reflects the overall performance of the model; **(c)** The decision curve (Net Benefit graph) evaluates the clinical application benefits of the model at different risk thresholds; **(d)** The confusion matrix intuitively displays the classification results of the optimal model on the test set, including the distribution of true positive, false positive, true negative and false negative.

For the specific performance analysis of the RF model (as shown in Fig 3), the model calibration curve (Fig 3a) shows good consistency between the predicted probability and the true probability. Although there is a certain deviation in the low probability interval, the overall trend is close to the perfect calibration curve. The ROC curve (Fig 3b) shows the classification ability of the model at different decision thresholds, and the AUC value is 0.6765, indicating that the RF model has certain advantages in distinguishing positive and negative samples. Decision curve analysis (Fig 3c) shows that the RF model yields a net benefit within a limited range of threshold probabilities; however, for a substantial portion of the threshold range, net benefit falls below zero. This indicates that clinical net benefit is confined to specific risk thresholds, and the model’s clinical applicability requires further validation in larger cohorts.. The confusion matrix (Fig 3d) shows that the RF model can accurately identify most negative samples (32/34), but there are certain deficiencies in the prediction of positive samples (2/7), suggesting that the model still has room for optimization in positive example recognition.

Overall, the RF model showed modest predictive performance in this exploratory study, providing reliable technical support for preoperative noninvasive prediction of CK19 expression. However, its recall rate and AUC value still have room for improvement, and future studies can try to further improve the model performance through feature optimization or ensemble learning methods.

### 3.4 SHAP visualization analysis

**Fig 4:**
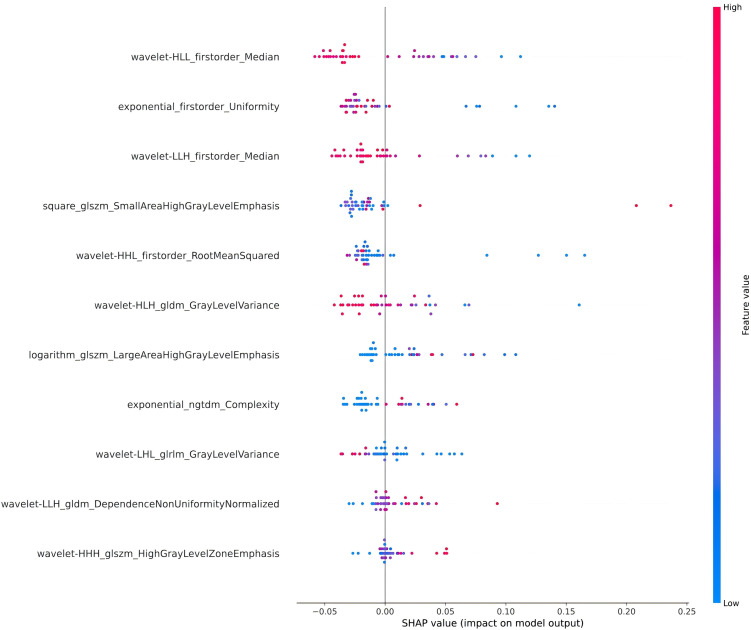
Visual analysis of the SHAP values of the RF model, showing the impact of important radiomics features on the model prediction results and their direction.

To further reveal the prediction mechanism of the random forest (RF) model and improve its clinical interpretability, this study used SHAP (SHapley Additive exPlanations) values to analyze and visualize the important features of the model. Based on the principle of game theory, the SHAP value can quantify the impact of each radiomics feature on the model prediction output, and use positive and negative values to indicate the direction of its action in driving the model output to increase or decrease. Fig 4 shows the SHAP value distribution of the RF model, which lists the 11 radiomics features that contribute most to the prediction results. The horizontal axis is the SHAP value corresponding to the feature, indicating the degree of influence of the feature on the prediction output; the vertical axis is the specific feature name. The color of the point represents the size of the feature value, red indicates a higher feature value, and blue indicates a lower feature value.

As can be seen from the figure, wavelet- HLL_firstorder_Median and exponential_firstorder_Uniformity are the two features that have the most significant impact on the model prediction, with a high SHAP value distribution. This indicates that the statistical characteristics of the tumor region, such as texture consistency and median, play a key role in distinguishing CK19 positive from negative expression. In addition, the features wavelet- LLH_gldm_DependenceNonUniformityNormalized and logarithm_glszm_LargeAreaHighGrayLevelEmphasis also show important contributions, which are mainly related to the density distribution and grayscale size area uniformity of the tumor region. It can be further observed from the color distribution that the high values (red) of some features (such as wavelet- HLL_firstorder_Median) mainly push the predicted value to move in the positive direction, suggesting that it has a higher feature value in samples with CK19 positive expression. The low values (blue) of another part of the features (such as exponential_firstorder_Uniformity) also have a positive impact on the prediction results, indicating that the reduction of these feature values may be associated with CK19 positivity.

## 4 Discussion

In this study, a noninvasive prediction model based on preoperative CT images was developed by combining radiomics with machine learning techniques to evaluate the expression of CK19 markers in patients with esophageal cancer. By extracting multidimensional radiomics features and screening out key features, this study effectively improved the prediction performance of the model. Comparison of multiple machine learning models showed that the random forest (RF) model demonstrated relatively higher accuracy and stability among the evaluated models, with an AUC value of 0.6765 and an accuracy of 0.8293. These findings suggest potential clinical utility in an exploratory context; however, given the modest recall for the positive class, direct clinical applicability remains to be validated in larger cohorts. Further SHAP value analysis provided the interpretability of the model and clarified the significant contribution of key features (such as wavelet- HLL_firstorder_Median and exponential_firstorder_Uniformity) to the prediction results. This prediction method combining high- throughput imaging features with machine learning provides a new perspective for traditional imaging diagnostic methods and has potential application value in preoperative individualized treatment decisions.

Random forest (RF) can be explained by its algorithmic advantages. By integrating multiple decision trees, RF can efficiently process high-dimensional data and reduce the risk of overfitting, thereby significantly improving the generalization ability of the model. Its built-in feature selection mechanism can automatically identify and prioritize important features and filter out irrelevant or redundant features, thereby further improving the accuracy and stability of the model. In addition, the robustness of RF enables it to maintain good prediction performance in the face of noise and missing data. In this study, the extraction of CT image features played a key role in the prediction performance of the model. The extracted features cover multiple dimensions such as shape features, first- order statistical features, gray-level co-occurrence matrix (GLCM), gray-level run length matrix (GLRLM), gray-level size zone matrix (GLSZM) and gray-level dependency matrix (GLDM), which help the model capture rich details in tumor images. In addition, wavelet transform further expands the feature space, enabling the model to effectively process information of different scales and levels, and improving the depth and accuracy of prediction. The most predictive features are selected through Lasso regression, which not only simplifies the model but also enhances its interpretability. Combining high-quality CT image feature extraction with an optimized feature screening strategy, RF demonstrated preliminary predictive potential in preoperative noninvasive prediction of CK19 markers.

To bridge the gap between quantitative imaging features and biological properties, it is essential to consider the pathophysiological basis of our findings. Cytokeratin 19 (CK19) expression is not merely a structural indicator but is intrinsically linked to tumor differentiation and invasiveness; elevated CK19 levels are often associated with more aggressive biological phenotypes and a higher risk of metastasis. Biologically, such aggressive tumors tend to exhibit significant microscopic heterogeneity due to rapid and irregular cellular proliferation, variations in vascular density, and focal necrosis. Our SHAP analysis revealed that features reflecting tissue complexity, particularly ‘Uniformity’ and ‘Median’, were dominant predictors. The observed correlation between lower textural uniformity and CK19 positivity suggests that the macroscopic heterogeneity captured by CT radiomics effectively serves as a surrogate marker for the internal biological disorganization of the tumor. The Random Forest model likely achieved superior performance because it could robustly model these complex, non-linear relationships between the imaging phenotypes of tissue heterogeneity and the invasive molecular phenotype represented by CK19 expression.

Traditionally, the expression of CK19 markers usually needs to be obtained through postoperative pathological examination or invasive biopsy methods, which not only increases the burden on patients, but also may delay the formulation of treatment decisions [14,15]. The prediction model based on CT radiomics developed in this study can evaluate the expression of CK19 markers non-invasively before surgery, providing a basis for patients’ staging, prognostic evaluation and treatment options. For example, high CK19 expression is usually associated with higher tumor invasiveness and metastasis risk. By identifying patients with high CK19 expression before surgery, clinicians can be guided to develop more active treatment strategies, such as expanding the scope of surgery, strengthening perioperative management or choosing more targeted adjuvant treatment options. In addition, this study achieved a quantitative evaluation of tumor microscopic characteristics by combining radiomics and machine learning technologies. This image-based molecular marker prediction method not only overcomes the limitations of traditional imaging diagnosis that relies on subjective experience, but also makes up for the deficiencies of molecular [16] pathology methods in time and space, laying a new technical foundation for personalized diagnosis and treatment [17]. More importantly, the interpretability analysis of the model (such as SHAP value) clarifies the contribution of specific imaging features to the prediction results, providing a scientific basis for clinicians to interpret the model. This transparent decision-making process helps to improve the acceptability and trust of the model in actual clinical applications [18,19].

Although this study shows that the preoperative noninvasive prediction model based on radiomics and machine learning has potential application value in evaluating CK19 expression in patients with esophageal cancer, there are still some limitations. First, this was a single‑center retrospective study with a modest sample size of 134 patients. The model was evaluated using a single 7:3 hold‑out split without external validation or cross‑validation, which limits the generalizability of the findings. Second, the test set contained only seven CK19‑positive cases, making performance metrics such as recall inherently unstable. The random forest model correctly identified only two of these seven cases, reflecting a recall of approximately 28.6% for the positive class, which is clinically inadequate and underscores the exploratory nature of this work. Third, the cohort exhibited substantial class imbalance, with CK19‑positive cases comprising only 17% of the sample. The high overall accuracy was largely driven by correct classification of the majority class, as reflected by a balanced accuracy of 0.613. No class‑balancing techniques were applied in this exploratory analysis. Fourth, we did not report 95% confidence intervals for performance metrics or perform formal statistical comparisons between models. Future work should incorporate bootstrap resampling to better quantify uncertainty. Finally, imaging data were obtained from two CT scanners using standardized protocols; however, heterogeneity in acquisition parameters across institutions may affect feature reproducibility. Multi‑center prospective validation is therefore essential before clinical translation. The lack of external validation is a limitation of this exploratory study. However, single‑center hold‑out split designs are acceptable for hypothesis‑generating radiomics research when interpreted cautiously [20–23]. Additionally, demographic and clinical data were not collected, as this study focused exclusively on CT‑derived radiomics features. Therefore, we could not assess potential confounding effects of clinical variables on model performance, which represents a limitation. Therefore, future studies need to improve its performance and clinical application value through multi-center large sample validation, combining multimodal imaging data, and optimizing the model structure.

## 5 Conclusion

This study successfully constructed a preoperative non-invasive prediction model based on radiomics and machine learning to evaluate the expression of CK19 markers in patients with esophageal cancer. By combining high-quality CT image feature extraction, Lasso feature screening, and comparison of multiple machine learning algorithms, the RF model performed best in terms of prediction performance and clinical applicability. The model interpretability analysis further revealed the contribution of key features to the prediction results, providing a scientific basis for the clinical application of the model. In the future, multi-center validation and multi-modal image fusion are needed to optimize the model performance and promote its widespread application in clinical practice.
